# Enhanced electronic transport properties of Te roll-like nanostructures

**DOI:** 10.3762/bjnano.13.106

**Published:** 2022-11-08

**Authors:** E R Viana, N Cifuentes, J C González

**Affiliations:** 1 Departamento Acadêmico de Física, Universidade Tecnológica Federal do Paraná, Campus Curitiba, 80230-901, Curitiba, Brazil; 2 Departamento de Física, Universidade Federal de Minas Gerais, 30123-970, Belo Horizonte, Brazil

**Keywords:** electrical characterization, field-effect transistors, hopping conduction, nanobelts, tellurium

## Abstract

In this work, the electronic transport properties of Te roll-like nanostructures were investigated in a broad temperature range by fabricating single-nanostructure back-gated field-effect-transistors via photolithography. These one-dimensional nanostructures, with a unique roll-like morphology, were produced by a facile synthesis and extensively studied by scanning and transmission electron microscopy. The nanostructures are made of pure and crystalline Tellurium with trigonal structure (t-Te), and exhibit p-type conductivity with enhanced field-effect hole mobility between 273 cm^2^/Vs at 320 K and 881 cm^2^/Vs at 5 K. The thermal ionization of shallow acceptors, with small ionization energy between 2 and 4 meV, leads to free-hole conduction at high temperatures. The free-hole mobility follows a negative power-law temperature behavior, with an exponent between −1.28 and −1.42, indicating strong phonon scattering in this temperature range. At lower temperatures, the electronic conduction is dominated by nearest-neighbor hopping (NNH) conduction in the acceptor band, with a small activation energy *E*_NNH_ ≈ 0.6 meV and an acceptor concentration of *N*_A_ ≈ 1 × 10^16^ cm^−3^. These results demonstrate the enhanced electrical properties of these nanostructures, with a small disorder, and superior quality for nanodevice applications.

## Introduction

The chalcogen tellurium (Te) is a rare element (0.002 ppm) in the Earth’s crust and a well-known p-type and narrow-bandgap (≈0.35 eV at room temperature) semiconductor material. Tellurium is widely used in thermoelectric devices, piezoelectric devices, photoconductive devices, gas sensing, nonlinear optical devices, solar cells, photonic crystals, holographic recording devices, radioactive cooling devices, field-effect transistors, infrared acousto-optic deflectors, and even for antifungal activity [1–7].

Several chemical and physical methods have been recently developed to synthesize Te-based nanostructures, such as monolayers (MLs), nanoparticles (NPs), nanorods (NRs), nanowires (NWs), nanobelts (NBs), nanotubes (NTs), nanoflowers (NFs) and chiral nanostructures [6–10]. Trigonal tellurium (t-Te) MLs have also been recently proposed as a silicon successor for nanoelectronics because of their high hole mobility and current density [3]. Combining these electrical properties with the facile synthesis of one-dimensional nanostructures may bring potential applications of this material in nanoscale optoelectronic integrated devices. However, only a few works have been dedicated to studying the electrical transport in Te-based one-dimensional nanostructures [11–14]. Te NTs have shown metallic character and decreasing electrical resistivity with temperature [11]. Te NWs encapsulated in boron nitride nanotubes have shown a large current-carrying capacity and p-type semiconducting characteristics, which can be reversed to n-type behavior after capping with Al_2_O_3_ [12]. Theoretical works have also demonstrated that field-effect transistors (FETs) with single n-type trigonal Te NWs outperform the ones built with three trigonal Te NWs [14]. One important group of nanostructures, different from hollow nanotubes and solid nanowires, is NBs. NBs have a well-defined geometry with a uniform rectangular cross section along their entire length. This particular morphology makes them a strong candidate for providing a thorough understanding of dimensionally confined transport phenomena, as presented in SnO_2_ NBs. Moreover, strain-induced polarization charges have been studied in p-type Te NBs [13].

In this work, we have studied the electronic transport properties of a distinct one-dimensional t-Te nanostructure with a roll-like morphology, which resembles cinnamon sticks. The nanostructures were obtained by a facile PVP-assisted hydrothermal route under mild conditions. A large quantity of these polycrystalline nanostructures with a diameter between 100 and 900 nm and a wall thickness around 50 nm were synthesized and characterized by scanning electron microscopy (SEM), energy dispersive X-ray spectroscopy (EDS), transmission electron microscopy (TEM), and selected-area electron diffraction (SAED). Individual Te roll-like nanobelts were connected in back-gate FETs and measured to characterize the electronic transport as a function of temperature.

## Methods

### Growth of Te roll-like one-dimensional nanostructures

Te nanostructures were grown via an environmentally friendly solvothermal method by the reduction of Na_2_TeO_3_ and passivation with polyvinylpyrrolidone (PVP), as reported elsewhere [15]. The procedure described by Wu et al. [15] for the synthesis of Te NWs was followed, with the modification of increasing the autoclave heating to 200 °C. All reagents used were of analytic grade, purchased from Sigma-Aldrich Chemicals Company, and directly used without further purification.

### Morphology, elemental analysis, and crystal structure

Morphology and elemental composition of the as-prepared products were characterized by scanning electron microscopy (SEM, FEI Quanta 3D FEG) at an acceleration voltage of 15.0 kV. An EDS system attached to the SEM was employed to analyze the chemical composition. TEM, high-resolution TEM (HRTEM) images, and SAED measurements were carried out in an FEI Tecnai G2-20 S-TWIN operated at 200 kV in a bright-field (BF) TEM mode. EDS point acquisitions were also performed during TEM analysis by using a silicon drift detector (SDD) from Oxford Instruments.

### Electronic transport

The electronic transport properties of the sample were investigated by acquiring and modeling the transfer and gate curves of Te roll-like single-nanostructure back-gate FETs, as well as the electrical resistivity of the nanostructures as a function of temperature from 5 to 400 K. The transport measurements were carried out in a low-noise custom-made system for electrical characterization of FET devices [16–18]. FET devices were built by laser writing optical lithography on 1 × 1 cm^2^ degenerate Si(100) substrates covered by a 300 nm thick high-quality SiO_2_ layer. A Cr(10 nm)/Au(100 nm) bilayer was thermally evaporated on the sample to produce good ohmic contacts (see Supporting Information File 1). This procedure follows the methodology developed for single-nanobelt back-gated FETs [16–19], also presented in Supporting Information File 1.

## Results and Discussion

**Figure 1:**
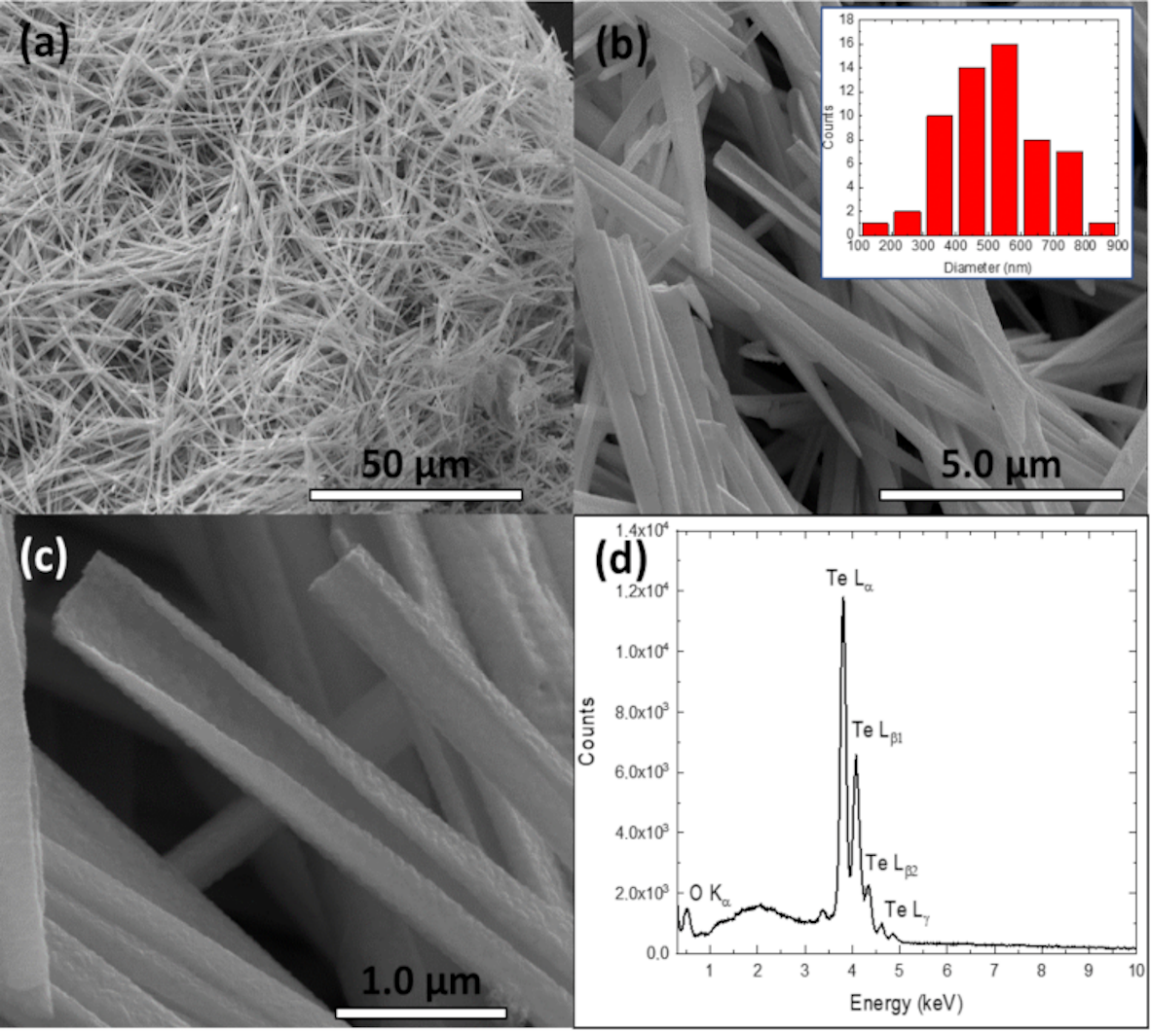
(a–c) SEM images at different magnifications of the roll-like Te nanostructures and (d) the EDS spectra of the sample. The inset of panel (b) shows the histogram of the diameter distribution of the nanostructures.

The morphology of the as-prepared Te roll-like one-dimensional nanostructures is shown in Figure 1. Figure 1a and Figure 1b display a representative overview of the nanostructures, which shows that the prepared samples are composed of large-scale roll-like one-dimensional nanostructures, comparable to the shape of cinnamon sticks. Most nanostructures exhibit one flat end and a tip at the other end. The size distribution of the nanostructures is shown in the inset of Figure 1b, with a mean diameter of around 550 nm. Due to the tip at the end of the nanostructures, the diameters were measured approximately at the center part, where the size is uniform. The wall thickness of the nanostructures was found between 45 and 55 nm.

Figure 1c shows a high-magnification SEM image of the nanostructures, which vividly demonstrates the surface rugosity and the lateral opening along the growth axis of the roll-like structure. Elemental analysis of the nanostructures is shown in Figure 1d, where the strong Te peaks and a residual oxygen peak in the EDS spectrum demonstrate the chemical composition of the nanostructures. This morphology has not been reported for the hydrothermal synthesis of Te nanostructures. Though, similar morphologies have been observed in Te scroll-like nanostructures grown by reflux processes [20] or Te shuttle-like scrolled nanostructures produced by amino acid-controlled hydrothermal growth [21]. However, the typical seed at the center of the scroll-like nanostructures grown by reflux processes was not observed in our case, nor was the characteristic sharp tip at both ends of the shuttle-like scrolled Te NTs.

**Figure 2:**
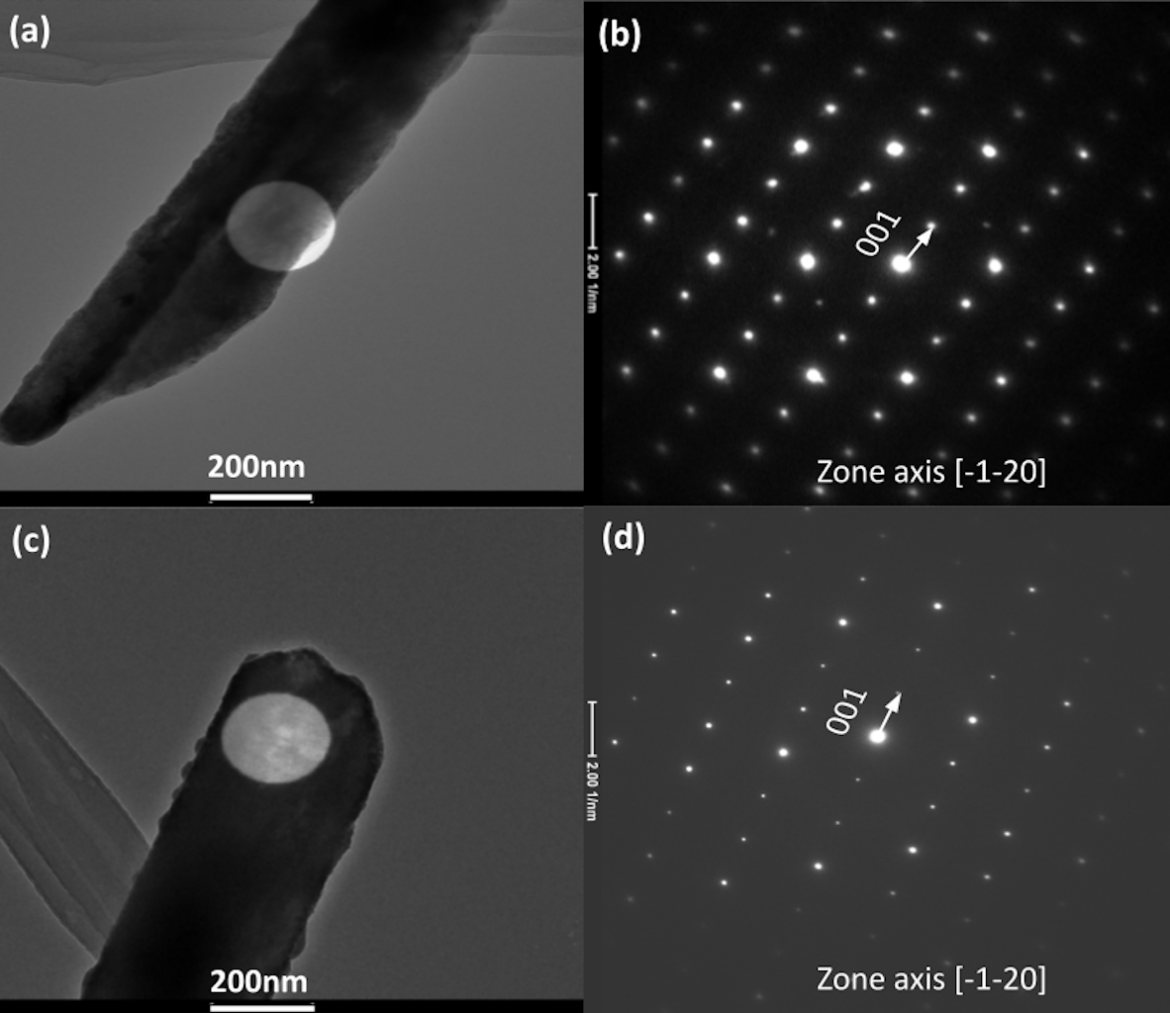
TEM image and SAED patterns of (a,b ) the flat end and (c, d) the tip of a roll-like t-Te one-dimensional nanostructure.

**Figure 3:**
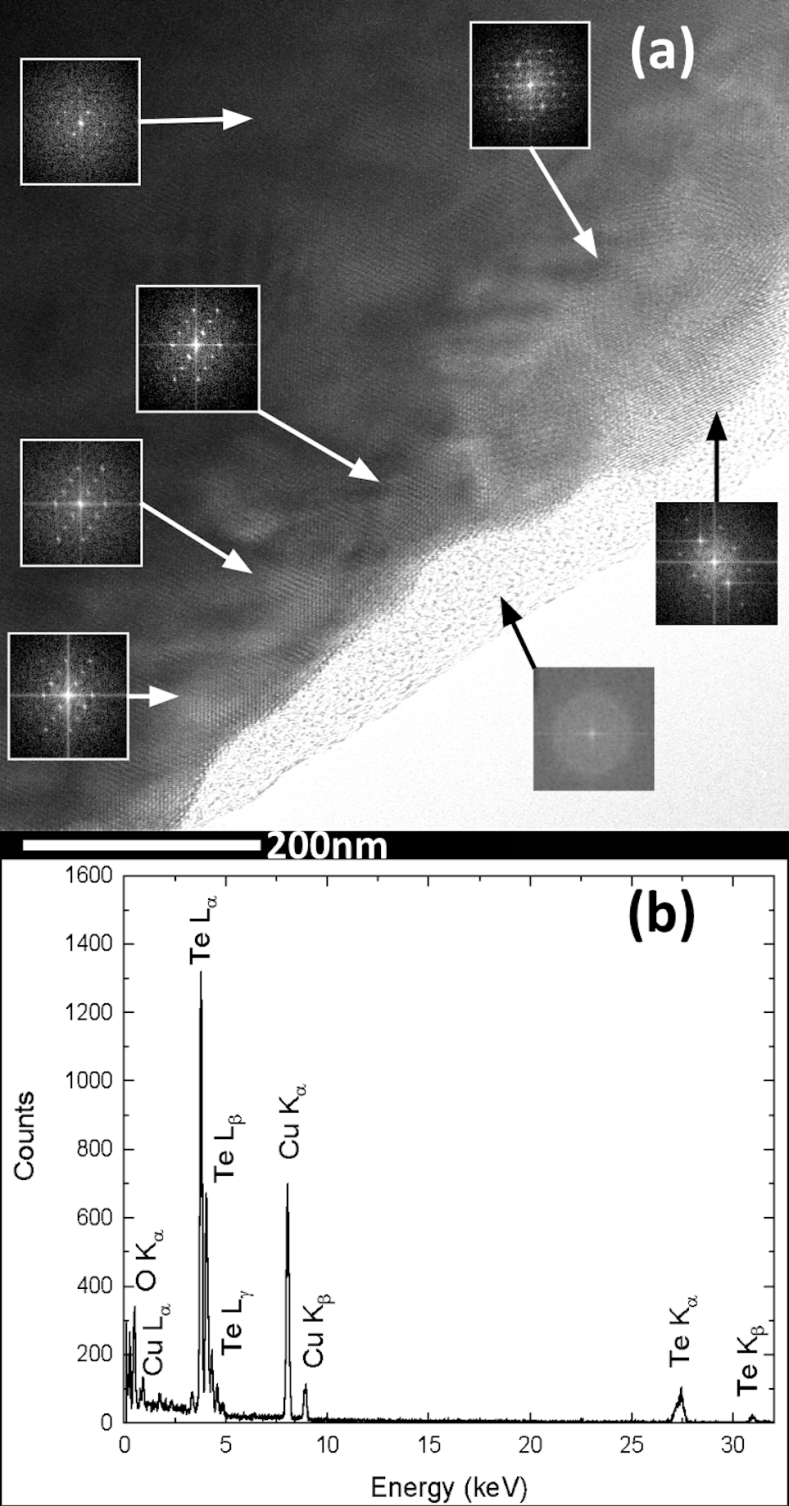
(a) HRTEM image of the roll-like t-Te one-dimensional nanostructure with FFT patterns from different regions of the sample. (b) EDS spectrum of the corresponding nanostructure.

Figure 2 shows TEM images and the corresponding SAED patterns of the roll-like Te nanostructures. The SAED patterns shown in Figure 2b and Figure 2d were acquired from the marked circular area in Figure 2a and Figure 2c, respectively (next to the tip of the nanostructure in Figure 2b, and next to the flat end of the nanostructure in Figure 2d). Detailed analysis of the SAED patterns, acquired along the [−1−20] zone axis, using CrysTBox software [22–23] demonstrates that the roll-like nanostructure is crystalline. The one-dimensional nanostructure has the trigonal crystal structure of bulk Te, growing in the [001] orientation (indicated by a white arrow in the SAED patterns) along the longitudinal direction (parallel to the axis of the Te nanostructure) and [210] growth toward the transverse direction (perpendicular to the growth axis). The [001] growth direction is usually observed in one-dimensional t-Te nanostructures and attributed to the anisotropy of the Te crystal structure [20–21]. However, a fast Fourier transform (FFT) analysis of different areas of the HRTEM images (see Figure 3a) shows that the nanostructures are polycrystalline, with well-oriented large grains and rotated small grains at the edges. Small amorphous areas were observed at the edges of the nanostructures. However, the well-defined SAED spot patterns of Figure 2b and Figure 2d, as well as the FFT patterns in Figure 3a are a clear indication of the crystalline nature of the nanostructures. The expected diffuse halo or broad circles associated with the diffraction of amorphous/nanocrystalline materials [24] were not observed. The EDS point analysis (Figure 3b) shows that nanostructures consist of Te, in agreement with the results of the SAED analysis and the above presented EDS analysis of the sample (Figure 1d).

**Figure 4:**
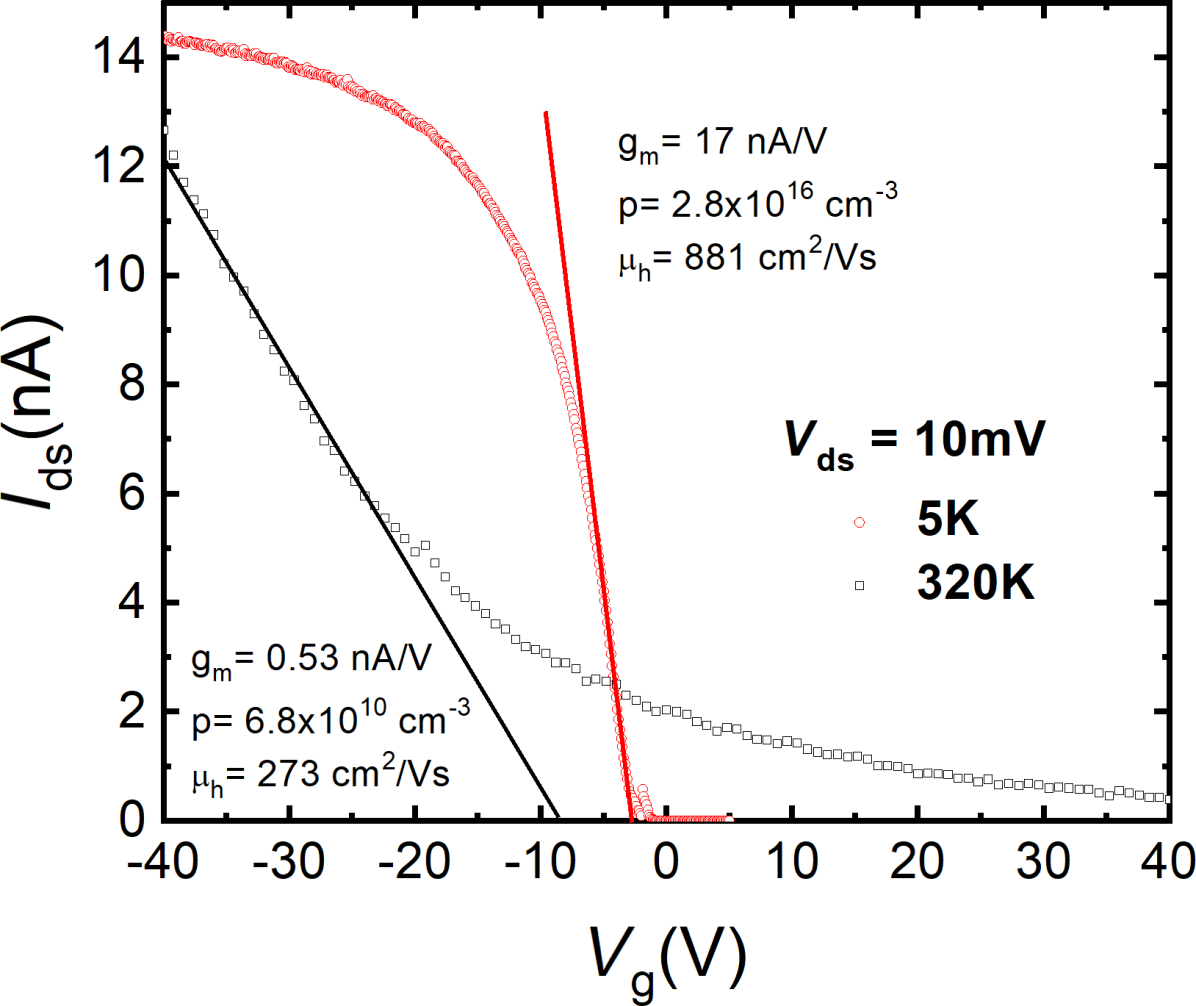
The transfer characteristic (*I*_ds_–*V*_g_) of a single roll-like t-Te NW-1 one-dimensional nanostructure back-gated FET acquired at 5 and 320 K.

The transfer characteristics of the t-Te NW-1 roll-like FET (*I*_ds_–*V*_g_ curve) at 320 and 5 K are shown in Figure 4 for *V*_ds_ = 10 mV. There is a strong increase of the transconductance (*g*_m_* = d*(*I*_ds_)/*d*(*V*_g_)) of approximately 30 times, from 0.53 nA/V at 300 K to 17 nA/V at 5 K. The p-type character of the NW is clear from these curves. Recent calculations of the band structure of t-Te have been revealed that the strong spin–orbit coupling breaks the fourfold degeneracy of the valence band at the H point of the Brillouin zone, creating two non-degenerated H4 and H5 bands and a doubly degenerated H6 band. The H4 and H5 bands contribute to the transport of free holes. However, the H6 band lies at lower energy, far from the Fermi level, and does not significantly contribute to electronic transport [25–26].

Field-effect hole mobility (μ_h_) and concentration (*p*) were estimated from the *I*_ds_–*V*_g_ curve using a parallel capacitor model for the FET [19,27]. This model has been successfully used to analyze the electronic properties of single semiconductor NWs of different cross sections and materials such as ZnO [27], SnO_2_ [16,19], and GaAs [17–18,28]. In this case, the calculations were done using μ_h_* = g*_m_*L*^2^/(*V*_ds_*C*_ox_) and *p =* 1/(*e*ρμ_h_), where *g*_m_* =* d*I*_ds_*/*d*V*_g_ is the transconductance, ρ is the resistivity, *C*_ox_ is the gate capacitance, and *e* is the electron charge. For a flat nanostructure, the gate capacitance can be obtained by a simple parallel-plate approximation, given by *C*_ox_ = ε_0_(ε_av_)*wL*/*d*_SiO2_ and ρ = (*R*·*w*·*t*/*L*). Here, ε_0_ is the permittivity of vacuum, ε_av_ = 1.95 is the averaged relative permittivity of the SiO_2_/air interface of the FET [19,29]. Also, *w* = 550 nm, *t* = 50 nm, and *L* = 5.97 µm are the diameter of the nanostructure, the thickness of the nanostructure, and the length of the FET channel, respectively. The thickness of the dielectric layer (SiO_2_) in the capacitor is *d*_SiO2_ = 300 nm.

Figure 4 shows the transfer curves (*I*_ds_–*V*_g_) of a single roll-like t-Te NW-1 one-dimensional nanostructure back-gated FET acquired at 5 and 320 K, as well as the calculated values of *g*_m_, *p,* and μ_h_. The hole mobility was estimated to be μ_h_(5 K) = 881 cm^2^/Vs and μ_h_(320 K) = 273 cm^2^/Vs, while the hole concentration was estimated to be *p(*5 K) = 8.8 × 10^10^ cm^−2^ and *p*(320 K) = 2.8 × 10^16^ cm^−3^.

**Figure 5:**
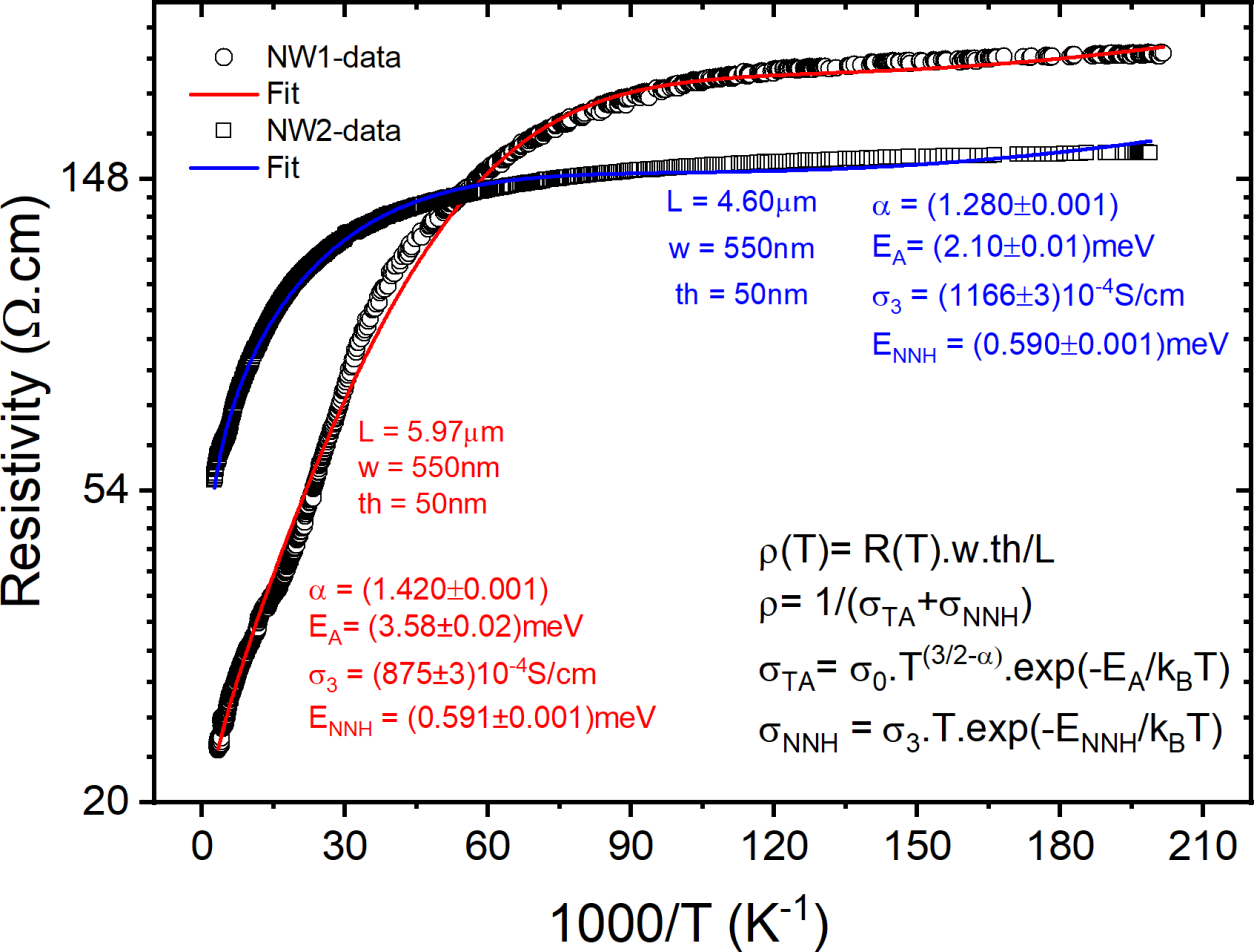
Resistivity as a function of temperature for the t-Te roll-like NW-1 and NW-2.

The measured electrical resistivity of NW-1 and NW-2, as a function of the temperature, from 300 down to 5 K, is shown in Figure 5. Two linear regions can be observed at high and low temperatures, corresponding to the thermally activated conduction (σ_TA_) of free holes and the nearest-neighbor hopping (NNH, σ_NNH_) [30–33], respectively.

In the high-temperature region, the thermally activated conduction can be expressed as [33]:


[1] $$\sigma_{\text{TA}}\left(T\right)=e\mu_{\text{h}}\left(T\right)p\left(T\right)$$

[2] $$\mu_{\text{h}}\left(T\right)\approx \mu_{0}T^{-\alpha }$$

[3] $$p\left(T\right)\approx N_{\text{V}}^{\text{eff}}T^{3/2}e^{-\frac{E_{\text{A}}}{k_{\text{B}}T}}$$

where µ_0_ is a constant. Since the mobility is mainly limited by phonon scattering at high temperatures, α is close to 3/2 [33–34]. *N*_v_^eff^ is the effective density of states of the valence band, *E*_A_ is the shallow acceptor ionization energy [33], and *k*_B_ is Boltzmann’s constant.

At lower temperatures, most of the free holes are recaptured by acceptors and cannot be thermally excited back to the valence band [30,33]. In this case, the thermally activated conduction of free holes becomes less important, and hole hopping directly between acceptor states in the impurity band becomes the primary conduction mechanism [30–33]. In this case, conduction is realized through NNH of charge carriers with small activation energy directly over impurity states. The conductivity in the NNH model is given by [35]:


[4] $$\sigma_{\text{NNH}}\left(T\right)=\sigma_{3}Te^{\frac{-E_{\text{NNH}}}{k_{\text{B}}T}}$$

[5] $$E_{\text{NNH}}=\frac{0.99e^{2}N_{\text{A}}^{1/3}}{4\pi \varepsilon_{0}\varepsilon_{r}}$$

where σ_3_ is a constant, *E*_NNH_ is the activation energy for NNH conduction, *N*_A_ is the acceptor concentration, and ε_r_ = 53.5 is the relative permittivity of t-Te [36].

Considering both TA and NNH conduction mechanisms, it is possible to extract some of the abovementioned parameters by fitting the resistivity data of NW-1 and NW-2 (Figure 5) with ρ(*T*) = 1/[σ_TA_(*T*) + σ_NNH_(*T*)]. This model explains well our NW-1 and NW-2 data over the whole temperature range. The ionization energy of shallow acceptors has not been reported for t-Te nanostructures. The ionization energy of shallow acceptors can be calculated to be 4 meV from the hydrogenic model [33] using the hole effective mass *m*_h_ = 0.91*m*_0_ for t-Te [37]. The theoretical value is in excellent agreement with our experimental values of *E*_A_ = 2.10 meV and *E*_A_ = 3.58 meV, found through the analysis of the resistivity data (Figure 5). These values are surprisingly small compared to the larger ionization energy of acceptors (near 1 eV) in semiconductors with small valence band effective mass [38–39]. However, the very large relative permittivity of t-Te, ε_r_ = 53.5, should also be considered.

The temperature dependence of the hole mobility in t-Te one-dimensional nanostructures has not been reported to the best of our knowledge. However, for bulk crystals and temperatures above 77 K, a power-law dependence of µ ~ *T*^−1.5^ [37] or weaker was described in the literature [34,40]. The power law strongly depends on the doping level of the t-Te crystals [34]. In our case, a weaker dependency with α = (1.420 ± 0.001) and (1.280 ± 0.001), see Equation 2, for NW-1 and NW-2, respectively, was found, demonstrating strong scattering by phonons in this temperature range.

At lower temperatures, the electronic conduction is dominated by NNH in the acceptor band with a low activation energy *E*_NNH_ ≈ 0.59 meV for both NWs. These values of *E*_NNH_ correspond to a concentration of acceptors of *N*_A_ ≈ 1 × 10^16^ cm^−3^ for both NWs. Due to the small acceptor ionization energy, the acceptor band will be completely ionized at high temperatures, and the acceptor concentration can be compared with the hole concentration found at 320 K (2.8 × 10^16^ cm^−2^). These values are in excellent agreement, corroborating our interpretation of the temperature dependence of the resistivity.

The temperature behavior of the resistivity of the t-Te roll-like nanostructures is somehow different than that previously reported for t-Te bulk crystals and NWs. The first striking characteristic of the previous reports is the metallic-like character of some bulk crystals [34,40–43] and NWs [44] at high temperatures, revealed by a decrease in the electrical resistance as the temperature drops. This behavior is not observed in our material, which exhibits semiconductor behavior over the whole investigated temperature range. At low temperatures, the electronic conduction in t-Te bulk material [45–46] and NWs [44] has been associated with variable-range hopping (VRH). VRH conduction of Mott (σ_M_ ~ exp(*T*_M_/*T*)^1/4^) and Efros–Shklovskii (σ_ES_ ~ exp(*T*_ES_/*T*)^1/2^) types has also been reported in chalcogenide semiconducting materials [29,31] and one-dimensional nanostructures [17–18,28,47]. However, VRH was not observed in our t-Te roll-like NWs, indicating low disorder in these nanostructures. Mobility values of more than 700 cm^2^/Vs have also been reported at room temperature for t-Te NWs [48]. However, in this case, the mobility was determined for elevated values of *V*_g_, apparently far from the linear region of the FET transfer characteristic. Other authors have reported t-Te NW hole mobilities between 72 and 349 cm^2^/Vs, for carrier concentrations in the range of 10^18^ cm^−3^ using the transconductance obtained from the linear region of the transfer characteristic of the FETs [49–51]. Our reported values are significantly higher at 320 K (273 cm^2^/Vs) and particularly at 5 K (881 cm^2^/Vs), but for lower carrier concentrations of approx. 1 × 10^16^ cm^−3^.

## Conclusion

We have demonstrated the facile synthesis of morphologically unique roll-like nanostructures. Extensive electron microscopy studies confirm that these nanostructures are made of pure and crystalline Te with a trigonal structure. The electronic transport properties of these nanostructures were investigated in a broad temperature range by fabricating single-nanobelt back-gated FET devices on SiO_2_/Si substrates. These nanostructures exhibit p-type conductivity with superior room temperature field-effect hole mobility compared to bulk and nanostructures of Te previously synthesized by other methods. The analysis of the temperature dependence of the electrical resistivity shows that, at high temperatures, the conduction occurs via free holes ionized from shallow acceptors with ionization energies between 2.10 and 3.58 meV, in agreement with the expected value from the hydrogenic model. Elevated free-hole mobility was also found (μ_h_(320 K) = 273 cm^2^/V·s, μ_h_(5 K) = 881 cm^2^/V·s) in these nanostructures. Thermoelectric devices, piezoelectric devices, photoconductive devices, gas sensing, solar cells, and field-effect transistors would have better performance if the mobility of charge carriers in the active region of the devices was greater. In addition, the low ionization energy of the defects found on these nanostructures also favors the control of doping and, consequently, the electrical properties of the nanowires. These superior quality transport properties demonstrate the potential use of t-Te roll-like nanostructures for electronic device applications.

## Supporting Information

File 1 Experimental section.
