# Supplementary material for: Enhanced electronic transport properties of Te roll-like nanostructures
Source: Beilstein J Nanotechnol. 2022 Nov 8;13:1284–91. doi: 10.3762/bjnano.13.106 (PMC9663975; doi:10.3762/bjnano.13.106)
Supplement: File 1 — Experimental section. [file Beilstein_J_Nanotechnol-13-1284-s001.pdf]

BEILSTEIN JOURNAL OF NANOTECHNOLOGY

# Supporting Information

for

# Enhanced electronic transport properties of Te roll-like nanostructures

E. R. Viana, N. Cifuentes and J. C. González

*Beilstein J. Nanotechnol.* **2022,** *13,* 1284–1291. doi:10.3762/bjnano.13.106

# Experimental section

License and Terms: This is a supporting information file under the terms of the Creative Commons Attribution License (https://creativecommons.org/licenses/by/4.0). Please note that the reuse, redistribution and reproduction in particular requires that the author(s) and source are credited and that individual graphics may be subject to special legal provisions.
The license is subject to the *Beilstein Journal of Nanotechnology* terms and conditions: (https://www.beilstein-journals.org/bjnano/terms)

## Te-NW FET device

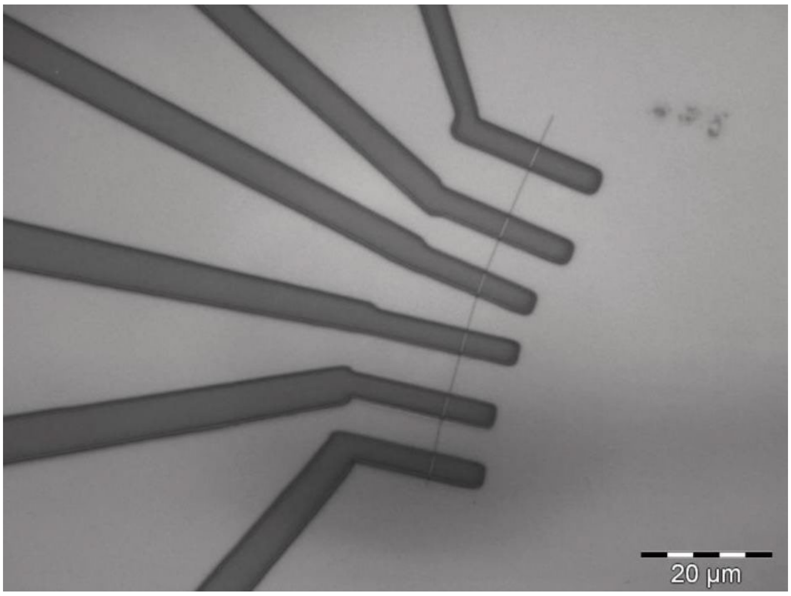


**Figure S1: Optical image of the FET device before Cr/Au metallization.**

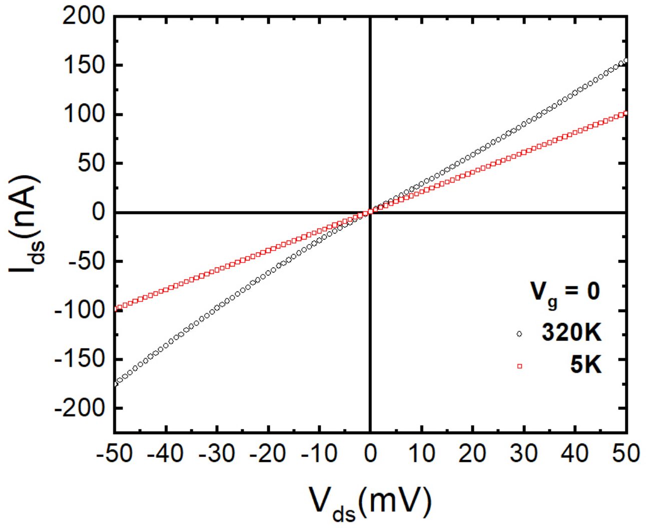


**Figure S2: $I_{ds}(V_{ds})$ curve of a Te roll-like nanostructure showing ohmic behavior.**

Due to the existence of potential barriers and other phenomena at the contacts, the linearity of the *I–V* curve in FETs and other electronic devices is usually disrupted at low voltages. This separation from the linear behavior is commonly stronger and more visible at low temperatures due to the suppression of thermal emission of the carriers over potential barriers at the contacts

or other thermally activation electronic transport mechanisms at the contacts. However, as shown in Figure S2 our *I–V* curves are highly linear at low and high temperatures. Therefore, the contacts of our devices are considered ohmic by the authors. The contact resistance ($R_c$) is another important issue. The ratio between the contact resistance to the intrinsic channel resistance ($R_i$) of the FET should be small in order to actually characterize the electrical properties of the channel. In electronic devices, the $R_c/R_i$ ratio increases at low temperatures. However, by measuring the resistance of channels of different lengths (in a transmission line configuration) we have estimated that the highest ratio in our devices is 17% at 5 K. We have considered that value low enough to extract valuable information from the devices.

For the extensive characterization presented here, the devices were submitted to thermal cycling between low and high temperatures. Only 22% of the devices survived this thermal stress, which is a good yield considering the difficulties in preparing functional FETs with nanowires of such complex morphology.

More effort should be dedicated to improving the yield of functioning devices and lowering the contact resistance ratio

## Optical Lithography

The authors performed standard optical lithography. Initially, Te nanowires are dispersed in deionized water. The solution is dripped onto one Si/$SiO_2$ substrate. On the substrate with isolated nanowires, a thin and homogeneous film of a light-sensitive polymer, that is, the photoresist, is dispersed. To control the thickness of the layer of photoresist, a rotating table with controlled speed (Spinner) is used. Subsequently, the photoresist solvent is evaporated, placing the substrate on a heated plate with a controlled temperature (Hotplate). The substrate covered by the photoresist is placed in a sample holder of the equipment where the optical lithography will be performed using a Microtech LaserWriter LW-405®. The LW is basically composed of an optical microscope, a UV Laser ($\lambda$ = 405 nm), and an XYZ motorized stage. The stage is controlled on the micrometer scale for the precise determination of the position of the nanowires on the substrate, and the positions where the UV laser will be applied, which will sensitize the photoresist. After defining in LW the XY positions of the edges of the substrate, manual searches are performed for the nanowires, obtaining the XY position of their ends and their respective size. A defined sufficient amount of nanowires (generally between four and six per substrate) is then passed to the layout, CleWin®, defining the edges of the

substrate, the insulated wires, and the places where you want to place the electrical contacts. After that, the regions drawn are exposed to the UV laser, sensitizing the photoresist in the desired locations. After exposing the film to the laser, we go through the process of removing the sensitized photoresist, that is, the development, using an appropriate solvent. After evaporating the metal, removal of the excess metal (lift-off) is carried out with a solvent for the non-sensitized photoresist. Therefore, the Cr/Au layer will be in the predetermined locations of the project, resulting in the single nanostructure back gate FET device.

## Summary

Our results demonstrate the elevated potential of Te roll-like nanostructures for electronic and optoelectronic applications, as well as the synthesis route and microfabrication of FET devices based on those nanostructures. The preparation of FET devices is complex due to the roll-like morphology of the nanowires, making the measurements of a large number of devices over the whole temperature range more difficult. Therefore, further work is needed to fully characterize electronic transport and its variations in these nanostructures.
